# Spontaneous Duodenal Perforation as a Complication of Kawasaki Disease

**DOI:** 10.1155/2015/689864

**Published:** 2015-03-26

**Authors:** Kambiz Masoumi, Arash Forouzan, Hossein Saidi, Hazhir Javaherizadeh, Ali Khavanin, Mohammad Bahadoram

**Affiliations:** ^1^Department of Emergency Medicine, Imam Khomeini General Hospital, Ahvaz Jundishapur University of Medical Sciences, Ahvaz 6193673166, Iran; ^2^Department of Emergency Medicine, Hazrate Rasoul Akram Hospital, Iran University of Medical Sciences, Tehran 14455364, Iran; ^3^Department of Pediatrics, Abouzar Children's Hospital, Ahvaz Jundishapur University of Medical Sciences, Ahvaz 6135715794, Iran

## Abstract

Kawasaki disease is generally known as a systemic vasculitis that often concerns doctors due to its serious cardiac complications; however, other visceral organs may get involved as well. Surgical manifestations of the intestinal tract in Kawasaki disease are rare. In this report, we describe the case of a 2.5-year-old boy with typical Kawasaki disease who presented with GI bleeding and surgical abdomen. The diagnosis of duodenal perforation was confirmed.

## 1. Introduction

Kawasaki disease (KD) is one of the most common vasculitis of childhood. It is a systemic inflammatory illness that particularly affects medium-sized arteries, especially the coronary arteries. Pathologic studies indicate that multiple organs and tissues are involved but long-term sequelae appear to occur only in the arteries. Possible etiologic factors include immunologic response, infections, and genetic factors [1]. Complications of Kawasaki disease primarily reflect cardiac Sequela, although noncardiac complications also may occur [2]. Vascular changes also can occur in peripheral and visceral arteries. Nonetheless, in rare cases virtually any vascular bed may be affected. Thus, case reports have included KD presenting as a cerebrovascular accident (e.g., acute encephalopathy, stroke, gastrointestinal obstruction, pseudoobstruction, or acute abdominal catastrophe) [3]. GI involvement from KD is not common. However, diarrhea, vomiting, abdominal pain, hepatic dysfunction, and hydrops of the gall bladder (GB) are relatively common, whereas pancreatitis, intestinal obstruction, bowel edema, and acute surgical abdomen are rare. This is a case report regarding acute surgical abdomen (duodenal perforation) due to KD.

## 2. Case Presentation

A 2.5-year-old boy with fever and erythematous rash on trunk and extremities was brought to the emergency department by his parents. Fever had started about 2 days ago, not relieved by cold compress and acetaminophen. Red eye associated with erythematous lips and oral mucosa appeared a day later. Then, erythematous rash was developed. Regarding his condition, amoxicillin, diphenhydramine, and acetaminophen were prescribed by a general physician in an outpatient clinic. Patient was brought to the emergency department because of unchanging condition in the evening of 3rd day.

On arrival, he seemed very irritated while crying. On examination, we found bilateral nonexudative conjunctivitis, erythematous maculopapular rash on trunk and extremities, oedema, erosion and erythema of lips and oral mucosa, and oedema and erythema of palms and soles. Pharyngitis, cervical, axillary, and inguinal lymphadenopathy were not detected. Heart and lung were normal. Abdomen was soft without tenderness and organomegaly. There was no peripheral cyanosis. Past medical history was negative for allergy. Parents mentioned loss of appetite of their son over the last few days. Patient was admitted with provisional diagnosis of Kawasaki disease. The next morning, diagnosis of Kawasaki disease remained unclear. Patient condition was the same as before and he received supportive care. Both results of primary laboratory studies and chest X-ray were normal. On the 5th day of fever, patient's condition remained unchanged. Therefore, based on fulfilled criteria for Kawasaki disease, intravenous immunoglobulin (IVIG) 20 gram/single dose was started (2 gram/kg/single dose over 12 hours, BW: 10 kg) in addition to aspirin 800 mg/day (80 mg/kg/day) in 4 divided doses. Fever and chills occurred following IVIG administration which was controlled by IV fluid therapy and transient discontinuation of IVIG. Then, IVIG was continued more slowly without any problem. Supportive care was continued and patient condition remained unchanged until his mother noted his cola coloured urine (on the 7th day). Immediately, aspirin was discontinued and some lab tests were ordered. Test results are shown in Table 1.

Glucose-6-phosphate dehydrogenase (G6PD) deficiency had been missed. Hydration, serum alkalization, packed cell transfusion (100 cc), and vitamin E were started. Dipyridamole 200 mg/day was started instead of aspirin. The next morning (8th day), other lab tests results came back which were as in Table 1.

At this time, echocardiography was done and normal findings were reported. Melena was reported by mother in the evening. Occult blood testing was obtained whose result was positive. Gastrointestinal bleeding, some specific foods, or medicine were the initial differential diagnosis of positive occult blood test in our case. Clinical manifestations gradually started to eliminate. Fever decreased (38.5). Oral and lips erosions were relieved. Palmar and plantar erythema were declined. Conjunctival congestion was decreased. In the next three days, fever was resolved, restlessness disappeared, appetite increased, and other manifestations were minimal.

Results of some laboratory tests (11th day) are shown in Table 1. On 11th day, regarding fever discontinuation, dipyridamole dose was changed to a 30 mg/day (3 mg/kg/day in 3 divided doses). Unexpectedly the patient vomited in the evening followed by restlessness, fever, and abdominal pain again. Overfeeding, complication of KD, or adverse effects of dipyridamole were suspected for patient symptoms including abdominal pain. Slow feeding and supportive care were recommended but abdominal exam showed tenderness with decreased bowel sound. Then, urgently, abdominal X-ray was ordered which revealed free subdiaphragmatic intraperitoneal air. Patient with diagnosis of acute abdomen underwent emergent laparotomy. Perforated ulcer was seen in anterior portion of descending duodenum. Pathology center reported degeneration and necrosis of the epithelial cells, inflammatory exudates in the tissue layers associated with thrombosis, and hemorrhage of the small submucosal arteries in duodenal specimen. He was discharged of pediatric surgery ward after ten days with good condition. Further investigations showed no cardiac involvement or other complications.

## 3. Discussion

Kawasaki disease (KD) is a systemic vasculitis, resulting in severe inflammation and necrosis of medium-sized vessels. It involves not only the coronary artery but also the vessels in many other systemic organs. Therefore, KD is a multisystemic disease and unusual symptoms may be part of the systemic illness.

Kawasaki disease is a clinical diagnosis. Although elevated creatinine levels, ESR, and platelets count may also be seen in limited patients [2], in this particular patient we observed CRP+++ by qualitative test and elevated ESR levels that may be because of IVIG infusion in addition to inflammation [4]. Also, hyponatremia (Na, 125 meq/L) means severe inflammation in KD [3], and it may be a clue to the diagnosis of KD [5].

The patient filled all diagnostic criteria of KD except for cervical lymphadenopathy, which is the least common diagnostic criterion [4]. Our patient went under standard treatment for possible diagnosis of typical KD. Following treatment, the patient underwent hemoglobin fall and hematuria on the 7th day. Despite the fact that IVIG therapy and taking aspirin can cause complications such as gastrointestinal bleeding in Kawasaki patients, studies show that their advantages in preventing cardiac complications are far more significant than their possible side effects [2, 6]. There are some causes regarding gross hematuria in KD including aspirin consumption in G6PD deficiency and following hemolysis, renal and urinary complication of KD, and IVIG complication. The risks of aspirin therapy appear to be similar to those reported in other settings, including chemical hepatitis with elevated transaminases, transient hearing loss, and, rarely, Reye's syndrome. However, these risks may be increased in patients with KD. Aspirin-binding studies have suggested that the hypoalbuminemia of children with KD predisposes them to toxic free salicylate levels despite measured (total) values within the therapeutic range [6]. Aspirin in G6PD deficiency in contraindicated and resulted in hemolysis and hematuria. In these cases, other anticoagulants replaced or IVIG only can be administered without use of aspirin. The efficacy of the latter needs further investigation [6]. With the exception of sterile pyuria, urinary abnormalities and renal disease are uncommonly associated with KD. Among the renal complications that have been noted in selected cases are acute interstitial nephritis, mild proteinuria, and acute renal failure. Hemolytic-uremic syndrome, immune complex-mediated glomerulonephritis, and acute interstitial nephritis, for example, have each been reported in a handful of cases [7]. IVIG may cause acute renal injury as well, especially preparations that use sucrose as a stabilizer [6].

Children with KD may present with a wide variety of gastrointestinal manifestations. Gastrointestinal tract involvement is not unusual in KD, and its prevalence is 2.3%. The clinical manifestations include vomiting, diarrhea, abdominal pain, abdominal distension, hepatomegaly, paralytic ileus, and hydrops of gallbladder [7, 8]. Diarrhea associated with vomiting and abdominal pain in 61% and vomiting alone in 44% of Kawasaki cases has been reported [9]. However, surgical manifestations are much less frequent and include small bowel obstruction and strictures, intussusception, ischemic colitis, perforation of duodenum, hemorrhagic enteritis, appendicitis, pancreatitis, bile duct stenosis, and hydrops of gall bladder. A case series from Italy reported 10 children ultimately diagnosed with KD who were treated for acute abdominal catastrophes including gallbladder hydrops and cholestasis, paralytic ileus, appendicular vasculitis, and hemorrhagic duodenitis. Acute surgical abdomen was the clinical presentation of KD in 4.6% of their patients [10]. Surgical abdominal complications of KD have been reported in the literature [3, 8–11].

Despite the studies conducted so far, the cause of early gastrointestinal involvement in some KD patients in not yet known. However, it is interesting to note that a selective expansion of circulating V*β*2 T cells has been demonstrated both in the blood and in the jejunal mucosa of patients with KD in the acute phase [12]. The GI mucosa, which may serve as a super antigen presenting organ, because of its large area and the abundance of major histocompatibility complex class II expression by epithelial/macrophages in the GI tract may be the portal of entry to these antigens. Immune system activation during the acute phase of KD is an important factor in pathogenesis of the disease. Selective increase in VB2+ T cells in small intestinal mucosa was found in one series of patients with KD [7, 11, 12]. One study showed various bacterial strains, mainly Gram positive cocci (streptococci and staphylococci), in jejunal biopsies of patients with acute KD compared with controls. This change in small bowel flora was not related to the severity of the GI tract involvement and manifested itself with diarrhea. Because staphylococcus enterotoxin and streptococcus exotoxin might serve as super antigens, the change in the small bowel flora in these patients might play a role in the pathogenesis of the disease [7, 12].

The typical pathology of the small intestine shows degeneration, necrosis, and desquamation of the epithelial cells, as well as inflammatory exudates in the lamina propria and in the muscular layer. Vasculitis and thrombosis of the small submucosal arteries or submucosa hemorrhage are frequently seen [7, 8]. Similar inflammatory changes involving the liver, pancreas, and the lymphoid tissue have also been described [3].

## Figures and Tables

**Table 1:** Laboratory test results in days 1, 7, 8, and 11.

Test	First day	7th day	8th day	11th day
HB	11 g/dL	6.5 g/dL	9.5 g/dL	11.5 g/dL
Plt	247 × 10^9^/L		293 × 10^9^/L	
WBC	12 × 10^9^ cell/L		8.4 × 10^9^ cell/L	6.9 × 9 cell/L (PMN: 54%/Eos: 12%/Lymph: 34%)
BS	150 mg/dL			
BUN	17 mg/dL		9 mg/dL	
Creatinine	0.6 mg/dL		0.4 mg/dL	
Sodium	125 meq/L		130 meq/L	
Potasium	4.1 meq/L		3.1 meq/L	
ESR	23 mm/hr		109 mm/hr	11 mm/hr
CRP	+		+++	
UA (Hb)		Trace	Negative	
Reticulocyte count		0.6%		
G6PD level		Deficient		
PT			10.2 sec	
PTT			38 sec	
ALT			51 U/L	
AST			79 U/L	
ANA			Negative	
RF			Negative	
Stool exam			Negative	
Stool culture			Negative	
Urine culture			Negative	
Blood culture			Negative	
